# Microseminoprotein-Beta Expression in Different Stages of Prostate Cancer

**DOI:** 10.1371/journal.pone.0150241

**Published:** 2016-03-03

**Authors:** Liisa Sjöblom, Outi Saramäki, Matti Annala, Katri Leinonen, Janika Nättinen, Teemu Tolonen, Tiina Wahlfors, Matti Nykter, G. Steven Bova, Johanna Schleutker, Teuvo L. J. Tammela, Hans Lilja, Tapio Visakorpi

**Affiliations:** 1 Prostate Cancer Research Center, Institute of Biosciences and Medical Technology (BioMediTech), University of Tampere, Tampere, Finland; 2 Fimlab Laboratories, Tampere University Hospital, Tampere, Finland; 3 Department of Pathology, Fimlab Laboratories, Tampere University Hospital, Tampere, Finland; 4 Prostate Cancer Research Center, School of Medicine, University of Tampere, Tampere, Finland; 5 Department of Urology, Tampere University Hospital, Tampere, Finland; 6 Departments of Laboratory Medicine, Surgery, and Medicine, Memorial Sloan Kettering Cancer Center, New York, United States of America; 7 Nuffield Department of Surgical Sciences, University of Oxford, Oxford, United Kingdom; 8 Department of Translational Medicine, Lund University, Malmö, Sweden; Florida International University, UNITED STATES

## Abstract

Microseminoprotein-beta (MSMB, *MSMB*) is an abundant secretory protein contributed by the prostate, and is implicated as a prostate cancer (PC) biomarker based on observations of its lower expression in cancerous cells compared with benign prostate epithelium. However, as the current literature on MSMB is inconsistent, we assessed the expression of MSMB at the protein and mRNA levels in a comprehensive set of different clinical stages of PC. Immunohistochemistry using monoclonal and polyclonal antibodies against MSMB was used to study protein expression in tissue specimens representing prostatectomies (n = 261) and in diagnostic needle biopsies from patients treated with androgen deprivation therapy (ADT) (n = 100), and in locally recurrent castration-resistant PC (CRPC) (n = 105) and CRPC metastases (n = 113). The transcript levels of *MSMB*, nuclear receptor co-activator 4 (*NCOA4*) and *MSMB-NCOA4* fusion were examined by qRT-PCR in prostatectomy samples and by RNA-sequencing in benign prostatic hyperplasia, PC, and CRPC samples. We also measured serum MSMB levels and genotyped the single nucleotide polymorphism rs10993994 using DNA from the blood of 369 PC patients and 903 controls. MSMB expression in PC (29% of prostatectomies and 21% of needle biopsies) was more frequent than in CRPC (9% of locally recurrent CRPCs and 9% of CRPC metastases) (*p*<0.0001). Detection of MSMB protein was inversely correlated with the Gleason score in prostatectomy specimens (*p* = 0.024). The read-through *MSMB-NCOA4* transcript was detected at very low levels in PC. MSMB levels in serum were similar in cases of PC and controls but were significantly associated with PC risk when adjusted for age at diagnosis and levels of free or total PSA (*p*<0.001). Serum levels of MSMB in both PC patients and controls were significantly associated with the rs10993994 genotype (*p*<0.0001). In conclusion, decreased expression of MSMB parallels the clinical progression of PC and adjusted serum MSMB levels are associated with PC risk.

## Introduction

Prostate cancer (PC) is the second most common cancer among men worldwide [1]. Measurements of prostate specific antigen (PSA) in blood are widely used to detect men at PC-risk and monitor men with PC. Due to low test-specificity at moderately elevated levels, indiscriminate use of PSA-testing results in large number of unnecessary biopsies and over-diagnosis with a consequent risk of overtreatment [2,3]. Hence, there is an urgent need for additional biomarkers that can provide enhanced specificity for the early detection of aggressive, lethal forms of the disease.

Microseminoprotein-beta (*MSMB*, MSMB aka MSP, PSP94) is one of the three most abundant proteins secreted by the prostate, along with PSA and prostatic acid phosphatase (PAP) [4]. Several studies have shown higher MSMB expression in normal and benign prostate tissue than in cancerous tissue [4–7]. Observations of higher MSMB expression in benign prostate tissue prompted some investigators to suggest that MSMB has a tumor suppressive role in PC [8]. Functional studies *in vivo* and *in vitro* also suggest a tumor suppressive role for MSMB [9]. Levels of MSMB in serum and urine have also been shown to be lower in PC patients than in controls [10]. Positive MSMB expression has been reported to associate with favorable prognosis in needle biopsies [11] and loss of MSMB staining has been shown to be associated with shorter time to biochemical recurrence in clinically localized PC [7]. By contrast, increased MSMB expression in prostatectomy specimens has been suggested to be associated with unfavorable disease outcomes [12]. Thus, the prognostic significance of MSMB expression in prostate tissue remains controversial.

It has been shown that the serum level of MSMB is associated with a single nucleotide polymorphism (SNP) in rs10993994 in the promoter region of *MSMB*, the gene encoding for MSMB [13,14]. The T allele of the SNP rs10993994 has also been associated with PC risk [15,16], which has been replicated in multiple studies [17–19]. Still, there is evidence of an association between low levels of MSMB in serum and increased risk of PC regardless of the rs10993994 genotype [20].

Published data on the regulation of *MSMB* expression by androgens are inconsistent. Some studies have shown that MSMB expression is independent of androgens [21,22]. However, Dahlman and co-authors [23] found that the *MSMB* transcript and MSMB protein were both significantly reduced after short-term androgen deprivation therapy (ADT). Enhancer of zeste homologue-2 (EZH2), a known epigenetic silencer of gene expression, has been suggested to silence *MSMB* expression in advanced PC [24]. EZH2 has also been shown to be overexpressed in late-stage PC [25].

The *MSMB* gene is located on chromosome 10q11.2 [26]. A read-through fusion transcript combining *MSMB* with adjacent gene nuclear receptor co-activator 4 (*NCOA4*) has recently been demonstrated by Nacu et al. [27] and confirmed in PC tissue and normal prostate tissue by Lou et al. [28]. They also reported that the *MSMB-NCOA4* fusion gene includes androgen response elements (ARE) suggesting that the fusion gene could be regulated by androgens. *NCOA4 (*aka *ARA70)* encodes an AR-associated protein that increases the transcriptional activity of AR in prostate cells [29]. It has been suggested that *MSMB-NCOA4* fusion could have a role in PC due to the important roles of *MSMB* in prostate tissue and of *NCOA4* as an enhancer of AR activity [27].

Due to the inconsistent data on the tissue expression of MSMB/*MSMB* in the prostate we wanted to evaluate protein expression by immunohistochemistry (IHC) in comprehensive cohorts of PC representing hormone-naïve localized and advanced disease, as well as locally recurrent castration resistant PC (CRPC) and CRPC metastases. Additionally, transcript levels of *MSMB*, *NCOA4* and *MSMB-NCOA4* were studied to evaluate the significance of the read-through fusion transcript. Finally, serum levels of MSMB as well as rs10993994 genotype were analyzed in a cohort of PC patients and controls.

## Materials and Methods

### Clinical tumor samples

Prostatectomy, needle biopsy and locally recurrent CRPC tissue specimens as well as serum samples were obtained from Tampere University Hospital (TAUH). The samples were de-identified and analyzed anonymously. The use of the prospectively collected samples was approved by the Ethical committee of the Tampere University Hospital and the written informed consent was obtained from the patients. The approval for use of retrospective collection of tissue samples without informed consent was obtained from the National Authority for Medicolegal Affairs according to the Finnish law. The use of CRPC metastases was approved by the Johns Hopkins Medicine Institutional Review Board, and written informed consent was obtained from the subjects. All samples used for IHC were formalin-fixed, paraffin-embedded (FFPE) samples. Tissue microarray (TMA) slides were created from the prostatectomy and CRPC samples.

#### Prostatectomy samples

MSMB expression was evaluated with IHC in 261 prostatectomy specimens. The characteristics of the cohort are shown in S1 Table. The same prostatectomy specimens have previously been analyzed for Ki-67 and EZH2 [25]. Disease progression was defined according to the blood PSA level, with biochemical recurrence (BCR) defined as a PSA level ≥ 0.5 ng/ml in two consecutive blood draws.

#### Needle biopsy specimens

The needle biopsy cohort consisted of 99 diagnostic needle biopsies from patients who subsequently received primary ADT. The Gleason score and treatment information are presented in S2 Table. The same specimens have previously been analyzed for Ki-67- and EZH2-immunohistochemistry [30]. Disease progression was defined by PSA measurements in two consecutive blood draws being 25% above nadir with an absolute increase of ≥2 ng/ml above nadir or development of new metastases [30,31].

#### Locally recurrent CRPC samples

Transurethral resection of the prostate (TURP) specimens of 105 men with evidence of disease progression during ADT. Treatment information is shown in S2 Table.

#### CRPC metastases

One hundred and thirteen CRPC metastases were collected from 32 patients who died of CRPC and underwent rapid autopsy. All patients had been treated with ADT. The same metastases have also been analyzed for Ki-67 and EZH2 [25].

#### Prostatectomy cohort for qRT-PCR analyses

Seventy-six freshly frozen prostatectomy specimens containing a minimum of 70% cancerous cells were used for qRT-PCR analysis. The characteristics of the cohort are presented in S1 Table. TRI-reagent (Molecular Research Center Inc., Cincinnati, OH, USA) was used to isolate total RNA from the freshly frozen clinical samples and cell lines according to the manufacturer’s instructions.

#### RNA-sequencing cohorts

Data from freshly frozen tumor material from 12 benign prostatic hyperplasia (BPH) samples, 28 hormone-naïve prostatectomy specimens and 13 CRPCs [32] as well as 301 samples, including 251 cancerous samples and 45 adjacent normal tissue samples from the Cancer Genome Atlas (TCGA)- cohort, were used for evaluating the expression levels of *MSMB*, *NCOA4* and *MSMB-NCOA4*.

### Immunohistochemistry

All FFPE-samples were stained with a monoclonal MSMB antibody (ab19070; Abcam, Cambridge, UK), and the prostatectomy- and CRPC-specimens were also stained with a polyclonal MSMB antibody (4).For the staining, Power Vision^+^ Poly-HRP IHC kit (ImmunoLogic, AD, Duiven, Netherlands) was used according to the manufacturer’s instructions. Briefly, samples were first deparaffinized and heat treated in pH 6 TriSodium citrate-buffer, containing 0.05% Tween. Sections were incubated overnight with the diluted primary antibody. For the mouse-derived commercial MSMB antibody, samples were blocked in post-blocking solution and were then incubated with poly-HRP-solution. UltraVision Detection System (Thermo Fischer Scientific Inc., Waltham, MA, USA) was used for the visualization of the bound primary antibody. Negative control without the primary antibody was included in all stainings. Slides were scanned with Aperio ScanScope XT scanner (Aperio Technologies, Inc., Vista, CA, USA). Scoring was performed in a blinded fashion using a virtual microscope (http://jvsmicroscope.uta.fi). Intensity and percentage of the stained tumor area was evaluated. Expression of MSMB was considered high when 20% or more of the tumor tissue was stained with an intensity of at least 1 on an intensity scale of 0–3. Mean values of Ki-67 defined low and high expression of Ki-67. EZH2 expression was defined as high when >50% of the tumor cells were stained.

### qRT-PCR for *MSMB*, *NCOA4* and *MSMB-NCOA4* and sequencing of *MSMB-NCOA4*

The SYBR Green assay (Thermo Fischer Scientific Inc., Waltham, MA, USA) was used according to the manufacturer’s protocol to detect the mRNA expression levels of *MSMB*, *NCOA4* and the *MSMB-NCOA4* fusion. The sequences for the primers were obtained from Lou et al. [28]. Thermal cycles were as follows: 95°C for 5 min., 95°C for 10 sec., annealing temperature 59°C (for *MSMB*), 62°C (for *NCOA4*) and 59.5°C (for *MSMB-NCOA4*) for 30 sec. and 72°C for 20 sec. The gene encoding the TATA- box binding protein (*TBP*) was used for normalization. Finally, PCR -products were separated on 1% agarose gels to confirm the specificity of the reaction. The *MSMB-NCOA4* fusion product was sequenced to confirm the presence of fusion. For the sequencing, PCR -products were extracted from the gel and purified with a QIAquick kit (QIAGEN Inc., Valencia, CA, USA) according to manufacturer’s protocol. Purified PCR-product was cloned using a TOPO-TA cloning kit (Life Technologies, Carlsbad, CA, USA). A BigDye terminator sequencing kit (Applied Biosystems, Foster City, CA, USA) was used for the sequencing reactions. In data analyses, median values were used as a limit between high and low expression of the mRNA levels of *MSMB*, *NCOA4* and *MSMB-NCOA4*.

### Genotyping the SNP rs10993994 in germ-line DNA and measurement of serum MSMB levels

Measurement of the serum MSMB levels and genotyping of rs10993994 were performed from 369 PC patients and 903 adult male age-matched (± two years) control subjects referred to the Department of Urology without evidence of PC. The characteristics of the cases are presented in S3 Table. The SNP rs10993994 was genotyped from the DNA samples using the Sequenom MassARRAY (Sequenom Inc., San Diego, CA, USA) system at the Institute for Molecular Medicine Finland (FIMM). Immunoassay measurements of MSMB were conducted on the AutoDelfia 1235 automatic immunoassay system (PerkinElmer, Waltham, MA, USA) at Dr. Lilja’s laboratory at Wallenberg Research Laboratories, Department of Translational Medicine, Lund University, Skåne University Hospital, Malmö, Sweden as previously described [20,33,34]. Free and total PSA were measured using the dual-label DELFIA Prostatus total/free PSA-Assay (PerkinElmer, Turku, Finland) [35] calibrated against the WHO 96/670 (PSA-WHO) and WHO 68/668 (free PSA-WHO) standards. All assay measurements were conducted with samples blinded to outcome.

### Statistical analyses

Fisher’s exact test, the Chi-squared test, the Mann-Whitney U-test and unpaired t-tests were used to analyze the association between MSMB, *MSMB*, *NCOA4* and *MSMB-NCOA4* expression and different clinico-pathological and expression variables. Paired t-tests were used to analyze differences between the expression levels of *MSMB*, *NCOA4* and *MSMB-NCOA4*. Kaplan-Meier analysis of progression free survival was used to analyze the prognostic significance of MSMB expression in different cohorts with the Mantel-Cox test. Fisher’s exact test and the Kappa-test were applied when comparing results obtained using the commercial MSMB antibody and those obtained with our polyclonal MSMB antibody. The Mann-Whitney U-test was used to compare the serum levels of MSMB and PSA between PC patients and controls. The data were analyzed with analysis of covariance (ANCOVA) to examine the association of PC and serum MSMB levels, adjusted for age at diagnosis and free PSA or total PSA. Both MSMB and PSA variables were log transformed. Logistic regression analysis was performed, also with SPSS, to study the association between the serum MSMB level and PC risk indicated by the Gleason score and the stage of PC. Spearman correlation was used to study correlation between free, total and free to total PSA levels and the MSMB level in blood of patients and controls. The Kruskal-Wallis test was used to analyze associations between serum MSMB level and the genotype of the SNP rs10993994. Spearman correlation was used to analyze correlation between the serum MSMB level and age at diagnosis.

## Results

### Comparison of the monoclonal and polyclonal MSMB antibodies

Prostatectomy and locally recurrent CRPC samples were stained with both a rabbit polyclonal MSMB antibody as described by Abrahamsson et al. [4] and a commercial monoclonal MSMB antibody (ab19070, Abcam, Cambridge, UK). Both staining intensity and area of cancerous tissue were assessed in scoring with MSMB expression defined as positive when 20% or more of the tumor area had staining intensity from 1 to 3. Staining results with the monoclonal and polyclonal antibodies were similar. Eighty percent of the prostatectomy samples manifesting low level MSMB expression stained with the polyclonal MSMB antibody also showed low level MSMB expression when stained with the monoclonal antibody (κ = 0.559, *p*<0.0001; Table 1). In CRPC samples, the staining results were even more consistent between polyclonal and monoclonal MSMB antibodies (κ = 0.77, *p*<0.0001; Table 1). Due to the consistent nature of the staining, all other samples were stained only with the monoclonal antibody.

**Table 1:** Comparison of a monoclonal MSMB antibody and polyclonal MSMB antibody. Prostatectomy and locally recurrent CRPC samples were stained with a rabbit polyclonal MSMB antibody (33) and a commercial monoclonal MSMB antibody (ab19070, Abcam, Cambridge, UK).

Variable	Polyclonal MSMB antibody		*p*	*κ*
	Low, n (%)	High, n (%)		
Prostatectomy specimens^1^^,^ ^2^				
Commercial monoclonal MSMB antibody: Low	122(80)	31(20)		
Commercial monoclonal MSMB antibody: High	12(18)	53(82)	<0.0001	0.559
CRPC specimens^1^^,^ ^2^				
Commercial monoclonal MSMB antibody: Low	82(99)	1(1)		
Commercial monoclonal MSMB antibody: High	3(27)	8(73)	<0.0001	0.77

^1^Fisher’s exact test

^2^Kappa-test.

### Prostatectomy specimens

**Fig 1:**
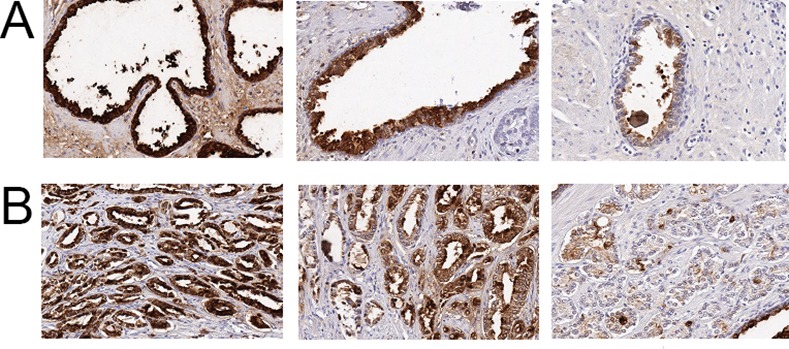
MSMB staining using a monoclonal MSMB antibody (ab19070, Abcam, Cambridge, UK). (A) High, medium and low MSMB staining in normal prostate tissue. (B) High, medium and low MSMB staining in prostate cancer tissue.

MSMB was heterogeneously expressed in the cytoplasm of the malignant prostate epithelium with distinct intensity differences observed in separate carcinomatous glands of the same specimen. Benign glands were commonly stained with high intensity, whereas cancerous glands only rarely showed high staining intensity and MSMB expression was commonly low (Fig 1). MSMB was expressed at a high level in 29% (75/261) of the cancerous tissue in prostatectomy specimens. Cancerous lesions had staining intensity of 0 in 89% (166/186) of the prostatectomy specimens, which was defined as low level expression of MSMB. MSMB expression tended to be inversely associated with the Gleason score but not with the Gleason grade (*p* = 0.0544 and 0.1669, respectively, Table 2). High MSMB expression was observed in 52/178 cases (29%) of the pT2 samples and 21/82 cases (26%) in pT3 samples (Table 2). There was no association between MSMB expression and diagnostic PSA levels or between MSMB expression and age (*p* = 0.8248; 0.2563, respectively; Table 2).

**Table 2:** Association of MSMB expression with clinicopathological variables and expression of EZH2 and Ki-67. These results are based on IHC-analysis of different cohorts.

Variable	MSMB expression			*p*
		Low	High	
Prostatectomy specimens n (%)	186 (71)		75 (29)	
Needle biopsy specimens n (%)	79 (79)		21 (21)	
Locally recurrent CRPCs n (%)	96 (91)		9 (9)	
CRPC metastases (patients) n (%)^1^	29 (91)		3 (9)	<0.0001
CRPC metastases (samples) n (%)	106 (94)		7 (6)	
Prostatectomy specimens:				
Gleason score, n (%)^1^				
≤6	60 (63)		36 (37)	
3+4 (7)	62 (73)		23 (27)	
4+3 (7)	33 (77)		10 (23)	
≥8	29 (83)		5 (15)	0.0544
Gleason grade, n (%)^1^				
2–3	126 (68)		60 (32)	
4	48 (77)		14 (23)	
5	8 (89)		1 (11)	0.1669
pT- stage, n (%)^2^				
pT2	126 (71)		52 (29)	
pT3	61 (74)		21 (26)	0.6562
PSA ng/ml (mean ± SD)^3^	14.1 ± 11.4		17.45 ± 30.6	0.8248
Age (mean ± SD)^4^	63.2 ± 0.4		62.4 ± 0.7	0.2563
EZH2 (mean ± SD)^3^	37.9 ± 24.0		32.4 ± 23.2	0.1040
Ki-67 (mean ± SD)^3^	11.1 ± 14.2		9.0 ± 11.6	0.2208
Needle biopsy specimens:				
Gleason score, n (%)^1^				
≤6	20 (83)		4 (17)	
7	33 (79)		9 (21)	
≥8	25 (76)		8 (24)	0.7869
T stage n (%)^2^				
T2	40 (80)		10 (20)	
T3	38 (78)		11 (22)	0.8097
PSA ng/ml (mean ± SD)^3^	5.4 ± 17.1		2.1 ± 2.4	0.3078
Age (mean ± SD)^4^	73.2 ± 0.79		71.4 ± 1.6	0.3169
Tumor area% (mean ± SD)^5^	30.5 ± 3.0		27.7 ± 5.6	0.6617
EZH2 (mean ± SD)^3^	24.3 ± 19.7		20.7 ± 18.6	0.4849
Ki-67 (mean ± SD)^3^	8.9 ± 0.7		7.6 ± 1.3	0.4055
Locally recurrent CRPCs:				
EZH2 (mean ± SD)^3^	56.8 ± 27.3		42.6 ± 26.3	0.1024
Ki-67 (mean ± SD)^3^	18.4 ± 14.7		11.1 ± 6.6	0.1545
CRPC metastases:				
EZH2 (mean ± SD)^3^	29.6 ± 24.9		36.5 ± 20	0.3295
Ki-67 (mean ± SD)^3^	11.5 ± 13.4		12.9 ± 7.5	0.2412

^1^χ^2^ test

^2^Fisher’s exact test

^3^Mann Whitney test

^4^Unpaired t test

^5^Unpaired t test with Welch correction. Low MSMB expression: <20% of the cancer tissue was positively stained (with intensity 1–3). High MSMB expression: ≥20% of the cancer tissue was positively stained (with intensity 1–3). EZH2 and Ki-67 were scored according to the stained area of the tumor tissue.

Although there was no significant association between MSMB expression and EZH2 expression, EZH2 expression tended to be higher in samples expressing low levels of MSMB (*p* = 0.1040; Table 2). There was no association between expression of MSMB and expression of the proliferation marker Ki-67 (*p* = 0.2208; Table 2) or with time to biochemical recurrence (*p* = 0.8997; S1 Fig).

### Needle biopsy specimens

Tissue sections of diagnostic needle biopsy specimens represented a cohort of older patients and/or more advanced disease at diagnosis than the prostatectomy cohort, and which received primary ADT. Twenty-one out of 99 (21%) of the biopsy samples expressed MSMB at a high level (Table2), with no association between MSMB expression and Gleason score or T-stage (*p* = 0.7869; 0.8097, respectively; Table 2). Similarly, there was no association between MSMB expression and the expression of EZH2 or Ki-67 (*p* = 0.4849 and 0.4055, respectively; Table 2). Further, cases with high compared to low MSMB expression showed no difference in time to biochemical progression by Kaplan-Meier analysis (*p* = 0.5621; S1 Fig).

### Locally recurrent CRPC specimens

MSMB expression was high in only 9% (9/105) of locally recurrent CRPC samples (Table 2), with no association between the expression of MSMB and EZH2 or Ki-67 expression (*p* = 0.1024; 0.1545, respectively; Table 2).

### CRPC metastases

High MSMB expression was observed in 9% of the cases (3/32) (Table 2). One out of these three patients had high MSMB expression in 1 out of 2 of his metastases. Another patient had high MSMB expression in 3 of 5 of the metastases, and the third patient in 3 of 4 of the metastases. MSMB was expressed at a high level in 6% (7/112) of all the metastases (Table 2). MSMB expression was not associated with the expression of Ki67 or with EZH2 (*p* = 0.2412 and 0.3295, respectively; Table 2).

### *MSMB*, *NCOA4* and *MSMB-NCOA4* fusion

**Fig 2:**
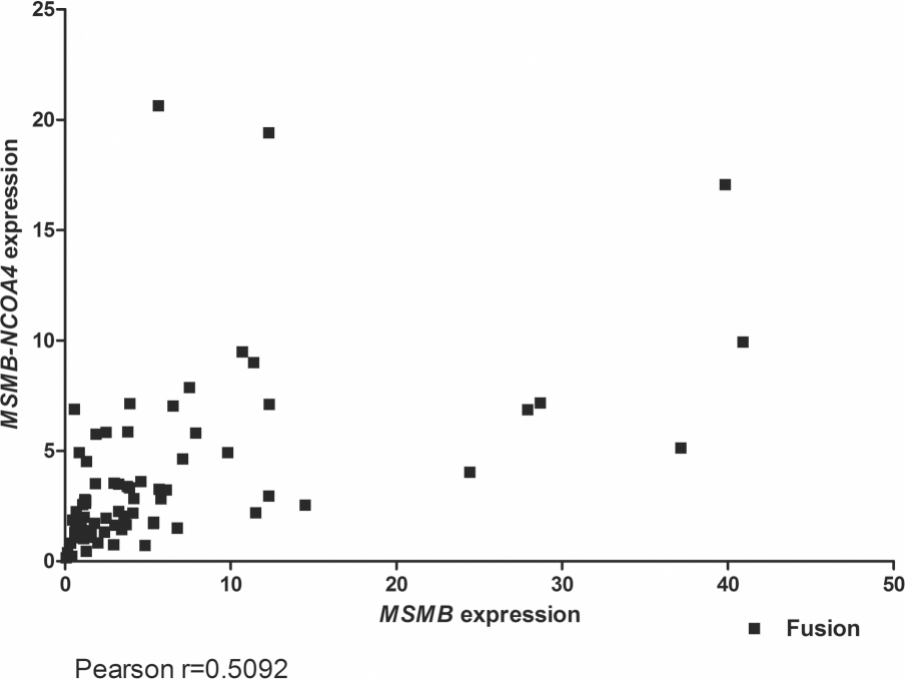
Correlation of *MSMB* and *MSMB-NCOA4* expression according to qRT-PCR. *MSMB* expression was positively correlated with expression of *MSMB-NCOA4*.

First, transcripts of *MSMB*, *NCOA4* and the fusion of *MSMB-NCOA4* were measured by qRT-PCR in RNA isolated from cancerous tissue in a cohort of patients with clinically localized PC treated with prostatectomy (n = 76). The data had a non-Gaussian distribution, with most samples showing below- average *MSMB* expression and only 25% (19/76) of samples manifesting MSMB-expression above average. *NCOA4* was expressed at a high level in 41% (31/76) of the samples and high levels of *MSMB-NCOA4* fusion was found in 32% (23/73) of the samples (Table 3). The expression of *MSMB-NCOA4* fusion transcripts was correlated with the expression of *MSMB* (Pearson r = 0.5092, *p*<0.0001; Fig 2). *MSMB* expression tended to be lower at higher tumor grade, but the expression levels of *MSMB*, *NCOA4*, and the *MSMB-NCOA4* fusion transcript were not significantly associated with the Gleason score (*p* = 0.0913; 0.2577; and 0.1963, respectively), pT-stage (*p* = 0.2760; 0.4819; and 1.000, respectively; Table 3), or time to biochemical recurrence (*p* = 0.3862; 0.4126; and 0.5940, respectively; S2 Fig).

**Table 3:** Association of *MSMB*, *NCOA4* and *MSMB-NCOA4* expression and the Gleason score, pT- stage and age. These results are based on Q-RT-PCR-analysis. Means of the normalized values were used as a cut-off value between high and low expression of *MSMB*, *NCOA4* and *MSMB-NCOA4*. Age refers to age at diagnosis.

Variable	*MSMB* expression		*p*	*NCOA4* expression		*p*	*MSMB-NCOA4* expression		*p*
	Low	High		Low	High		Low	High	
Prostatectomy specimens (%)^1^	57 (75)	19 (25)		45 (59)	31 (41)		50 (68)	23 (32)	0.0003
Gleason score, n (%)^2^									
≤6	24 (86)	4 (14)		16 (57)	12 (43)		22 (73)	8 (27)	
7	18 (62)	11 (38)		20 (69)	9 (31)		15 (56)	12 (44)	
≥8	10 (83)	2 (17)	0.0913	10 (83)	2 (17)	0.2577	9 (82)	2 (18)	0.1963
pT- stage, n (%)^3^									
pT2	30 (68)	14 (32)		24 (55)	20 (45)		30 (70)	13 (30)	
pT3	22 (81)	5 (19)	0.2760	19 (63)	11 (37)	0.4819	19 (68)	9 (32)	1.000
Age (mean ± SD)^4^	62.0 ± 0.7	61.5 ± 1.6	0.7511	61.7 ± 0.9	61.8 ± 0.8	0.9071	61.9 ± 0.8	61.8 ± 1.3	0.9089

^1^ One-way analysis of variance

^2^χ^2^ test

^3^Fisher’s exact test

^4^Unpaired t test.

**Fig 3:**
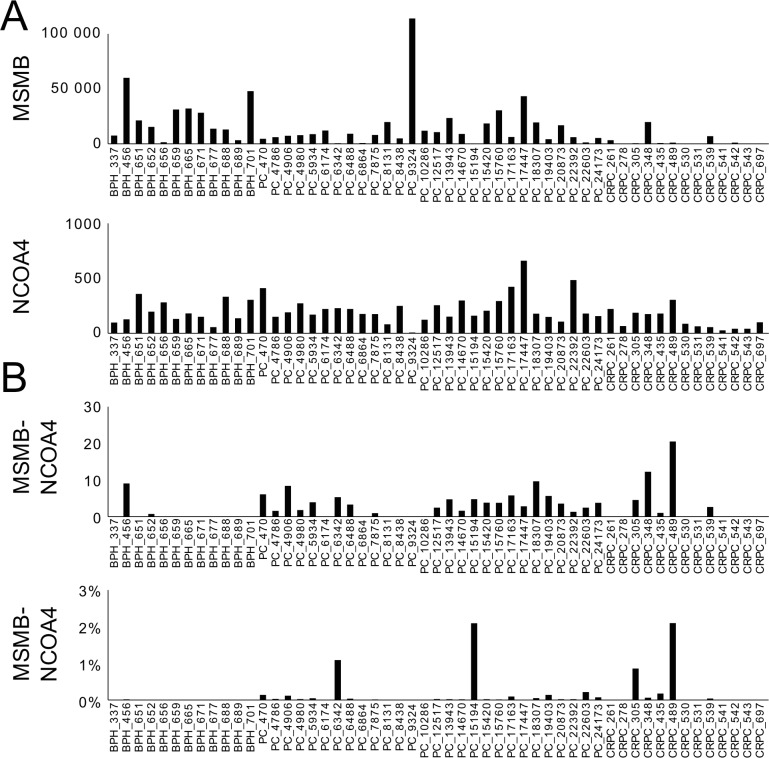
RNA-sequencing data of expression of *MSMB*, *NCOA4* and *MSMB-NCOA4* in BPH, PC and CRPC samples. (A) *MSMB* was expressed at higher level than *NCOA4*. (B) Lower panel shows the percentage of the *MSMB-NCOA4* reads from the total reads of *MSMB* and *MSMB-NCOA4*. *NCOA4* was expressed at lower level than *MSMB*. The fusion gene expression portion was 2.2% at highest, out of the sum of MSMB and MSMB-NCOA4 reads. Y-axis represents normalized read count.

Second, the detection of *MSMB*, *NCOA4* and *MSMB-NCOA4* fusion transcripts was assessed using RNA-seq in two cohorts; one containing 12 BPH, 28 hormone-naïve PC, and 13 CRPC samples [32], as well as a second TCGA-cohort consisting of 301 prostate adenocarcinoma tumors. Overall, the expression of *MSMB-NCOA4* was low and expression was observed more frequently in PC than in BPH tissue, although low levels of expression were detected in 2 of the 12 BPH samples (Fig 3). In the sample that expressed *MSMB-NCOA4* at the highest level, only 2.2% of the transcripts starting from the *MSMB* promoter formed a transcript with *NCOA4* (Fig 3B). *NCOA4* was expressed at a significantly lower level than *MSMB* in both cohorts (Fig 3 and S3 Fig).

### Serum MSMB levels and SNP rs10993994

There was no significant correlation between MSMB levels in serum and age at diagnosis of PC patients (Spearman r = 0.1550) or age at blood draw among controls (Spearman r = 0.1876; S4 Fig). In PC cases, the median level of MSMB in serum (23.0 ng/ml; interquartile range (IQR) 15.9) was not different (*p* = 0.2422) from that in controls (21.8 ng/ml; IQR 15.3; Table 4). However, when adjusted by age and free or total PSA at diagnosis, there was a significant association between PC risk and the serum levels of MSMB in the covariance analysis (*p*<0.001; Table 5). MSMB levels were higher in controls (the mean MSMB level was 29.7 ng/ml when adjusted by age and total PSA at diagnosis and 29.2 ng/ml when adjusted by age and free PSA at diagnosis) compared to PC patients (the mean MSMB level was 21.1 ng/ml when adjusted by age and total PSA at diagnosis and 22.4 ng/ml when it was adjusted by age and free PSA at diagnosis) (Table 5). Further, the serum MSMB level was associated with risk indicated by the Gleason score and with clinical stage at diagnosis (S4 Table), with the association remaining significant in non-metastatic or metastatic cases alone. Additionally, PC patients had a significantly lower free/total PSA ratio compared with controls (*p*<0.0001; Table 4). There was a statistically significant albeit weak positive correlation between levels of MSMB and free PSA as well as between MSMB and total PSA in serum from cancer cases (Spearman r = 0.3990 and 0.3200, respectively) and in controls (Spearman r = 0.3421 and 0.2710, respectively), *p*<0.0001 in all (S5 Fig), and significant association between the free-to-total PSA ratio and the serum levels of MSMB in cases and controls (Spearman r = 0.2142; r = 0.1351, respectively), *p*<0.0001 in all (S5 Fig).

**Table 4:** Characteristics of PC patients and controls of blood sample cohort.

Biomarker			
	Mean (±SD) case subjects / control subjects	Median (IQR) case subjects / control subjects	*p*
MSMB (ng/ml)^1^	27.8 (± 24.4) / 26.5 (± 24.7)	22.9 (15.9) / 21.8 (15.3)	0.2422
Free/ Total PSA (ng/ml)^1^	17.4 (± 11.4) / 28.4 (± 12.8)	14.7 (9.15) /26.4 (15.7)	<0.0001
Age^1^^,^ ^2^	68.1 (± 8.3) / 64.6 (± 9.3)	68 (10.7) / 64 (11)	<0.0001

^1^Mann Whitney test

^2^Age of the cancer cases refers to age at diagnosis and age of the controls refers to age at the time when the blood sample was taken.

**Table 5:** Characteristics of PC patients and controls of blood sample cohort adjusted by covariance analysis.

Characteristic	Cases		Controls		
	Mean	SD	Mean	SD	p***
Age at diagnosis	68.1	0.5	64.6	0.3	<0.001
MSMB *	21.1	1.5	29.7	0.9	<0.001
MSMB #	22.4	1.4	29.2	0.9	<0.001
Total PSA **	22.5	3.1	4.6	1.9	<0.001
Free PSA **	3.6	0.4	1.2	0.3	<0.001
Free to total PSA **	0.2	0.01	0.3	0.004	<0.001

* means adjusted by covariance analysis for age at diagnosis, total PSA (log2-transformed)

# means adjusted by covariance analysis for age at diagnosis, free PSA (log2-transformed)

** means adjusted by covariance analysis for age at diagnosis, MSMB (log2-transformed)

*** F test of case-control differences from covariance analysis.

Among the PC patients, the MSMB levels in serum were not associated with Gleason score in prostatectomy or biopsy specimens (*p* = 0.6621; 0.819, respectively; Table 6), but serum MSMB levels and pT-stage (*p* = 0.0288) and age at diagnosis (*p* = 0.0452) tended to be weakly associated in prostatectomy cases (Table 6). Kaplan-Meier analysis showed no association between the serum MSMB level and time to biochemical recurrence in patients treated with prostatectomy or radiation therapy (*p* = 0.1396; 0.1925, respectively; S6 Fig) or hormone-treated patients (*p* = 0.2070; S6 Fig).

**Table 6:** Association of serum MSMB with the Gleason score, pT-stage and age at diagnosis.

Variable	Level of serum MSMB		*p*
	Low, n (%)	High, n (%)	
Gleason score (biopsy), n (%)^1^			
≤6	82(50)	82(50)	
7	55(48)	59(52)	
≥8	33(53)	29(47)	0.8194
Prostatectomy-treated patients			
Gleason score, n (%)^1^			
≤6	15(44)	19(56)	
7	37(54)	32(48)	
≥8	8(50)	8(50)	0.6621
pT- stage, n (%)^2^			
pT2	37(44)	47(56)	
pT3	24(67)	12(33)	0.0288
Age (mean ± SD)^3^	67 ± 7.5	69 ± 8.9	0.0452

^1^Chi-square test

^2^ Fisher’s exact test

^3^Unpaired t test, Median (Gleason score: biopsy 23.5 ng/ml; prostatectomy: 21 ng/ml, pT-stage: 20.9 ng/ml) was used as a limit between low and high expression of MSMB.

**Fig 4:**
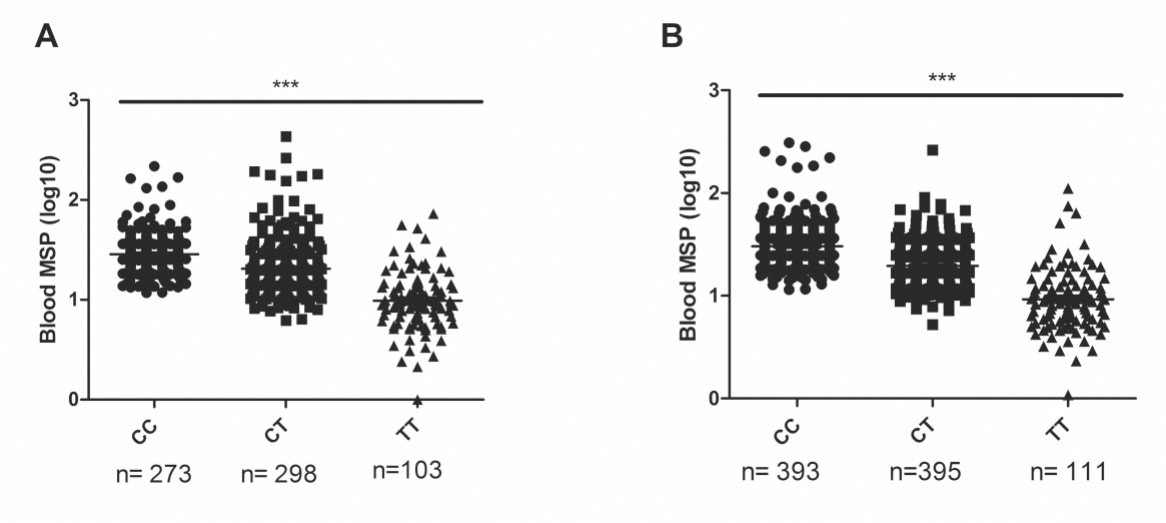
Genotype of SNP rs10993994 and MSMB level in blood of (A) PC patients and (B) controls. Lower MSMB level was associated with T-allele of the SNP rs10993994. Kruskal-Wallis test was used to compare the different groups. Lines represents the mean MSMB level of the different genotypes. *** denotes *p*<0.0001.

In contrast, there was a very strong association between low MSMB levels in serum and TT risk genotype of the SNP rs10993994 of both cancer cases and controls (*p*<0.0001; 0.0001, respectively; Fig 4). However, the SNP rs10993994 was not associated with Gleason score at biopsy or prostatectomy specimens or with pT-stage at prostatectomy (*p* = 0.7959; 0.4683; 0.3584, respectively; Table 7). Although the frequency of TT genotype tended to be slightly higher in cancer cases (15%) compared with controls (12%), there was no association of the rs10993994 genotype and cancer risk in this cohort (*p* = 0.5114; Table 7).

**Table 7:** Association of the SNP rs10993994 with the Gleason score of the biopsy and prostatectomy specimens and pT-stage.

Variable	rs10993994 genotype			
	CC	TC	TT	*p*
Cancer cases, n (%)^2^	154 (42)	160 (43)	54 (15)	
Controls, n (%)	394 (44)	396 (44)	111 (12)	0.5114
Gleason score (biopsy), n (%)^1^				
≤6	64 (41)	73 (46)	21 (13)	
7	49 (43)	49 (43)	16 (14)	
≥8	25 (40)	25 (40)	12 (20)	0.7959
Prostatectomy-treated patients				
Gleason score, n (%)^1^				
≤6	11 (32)	17 (50)	6 (18)	
7	31 (45)	31 (45)	7 (10)	
≥8	9 (56)	6 (38)	1 (6)	0.4683
pT- stage, n (%)^2^				
pT2	5 (42)	7 (58)	0 (0)	
pT3	42 (41)	46 (45)	14 (14)	0.3584

^1^Chi-square test

^2^Fisher’s exact test.

## Discussion

In this paper, we studied the potential role of MSMB/*MSMB*/*MSMB-NCOA4* as PC biomarkers in cohorts corresponding to different stages of PC from early to advanced disease. We showed that MSMB expression is reduced in more advanced PC, being lowest in the CRPC. The low MSMB protein levels in CRPC were consistent with our RNA-seq data indicating low *MSMB* transcript levels in the CRPC compared to PC samples. The finding of decreased expression of MSMB/*MSMB* in PC compared with non-malignant prostate, as well as association of the loss of expression with more advanced and higher-Gleason-score disease, is consistent with several previous publications [5–7]. However, CRPC cases have not been thoroughly studied before. Dahlman et al., [23] studied MSMB expression in 3 CRPC metastases and found low *MSMB* transcript levels. Here, we showed that both transcript and MSMB protein levels are low in CRPC with only 9% of the CRPC cases expressing MSMB.

In our study, MSMB protein or mRNA levels were not associated with prognosis in prostatectomy or ADT- treated patients. Some previous publications have shown an association between low MSMB and poor prognosis in PC [7,11], whereas others demonstrated high MSMB or *MSMB* transcript levels and poor prognosis [12,36]. There may be several reasons for the discordant findings. Patient cohorts vary. Here, we used three cohorts: **(a)** a prostatectomy cohort of 261 cases with a mean follow-up of 7.3 years, **(b)** a prostatectomy cohort of 76 cases with a mean follow-up of 5.2 years and **(c)** a cohort of primarily ADT-treated patients with a mean follow-up of 3.6 years. Cohorts a and c were used for immunohistochemical detection of MSMB protein and cohort b for qRT-PCR measurement of *MSMB* transcript. Consistently, none of the cohorts showed prognostic value for MSMB/*MSMB*. One putative variable is the antibody used for immunostaining. We tested two commonly used antibodies, a monoclonal antibody and a polyclonal one. They gave highly concordant staining patterns. Thus, the antibodies are likely not the cause of the discrepant data.

A read-through fusion transcript of *NCOA4* and *MSMB* has recently been demonstrated in some prostate cancers [27,28]. Thus, we aimed to measure the expression of the fusion transcript in clinical specimens. We found that *MSMB*-*NCOA4* expression was correlated with *MSMB* expression and not associated with the prognosis of prostatectomy-treated patients. Based on RNA-seq data, the expression level of the *MSMB-NCOA4* fusion was also very low, even in the sample expressing the fusion transcript at the highest level. Thus, the clinical relevance of the fusion gene remains unknown. Read-through transcripts are relatively common in cancer, but often their functional significance remains unknown [37].

Beke et al. [24] have suggested that *MSMB* is a target for EZH2. They showed that *MSMB* transcription is diminished by EZH2 via tri-methylation of H3K27. We studied the association between the expression of MSMB and EZH2 in prostatectomy, needle biopsy and CRPC samples and saw a slight trend of higher EZH2 expression in samples where MSMB expression was low (Table 2). However, no significant association was observed. Thus, it is likely that other mechanisms lead to decreased expression of MSMB in CRPC.

We did not found a significant difference in serum MSMB levels between the cancer cases and the controls when the comparison was performed without adjustment with age and free or total PSA. In fact, an average MSMB level was slightly higher in sera of cases compared to controls. However, when adjusted for age at diagnosis and free or total PSA, the serum MSMB level was higher in the blood of controls than in PC patients. This result is consistent with those obtained in the study by Haiman et al. [20].

CT and TT genotypes of the SNP rs10993994 were associated with lower MSMB level in serum both in PC cases and controls, consistent with previous studies [13,14,20,38]. In the study by Xu et al. [14], the T allele of rs10993994 was also associated with PC risk. In our study we did not see the association. More studies are warranted to investigate the potential role of serum MSMB and rs10993994 genotype in detection of PC.

In conclusion, the study confirms that MSMB expression is reduced in PC and is lowest in CRPC. The low serum level of MSMB was also associated with the risk of PC when adjusted for age and PSA. In the future, it will be important to study whether MSMB could be used together with other biomarkers to detect clinically significant PC. In addition, new reagents to detect different forms of MSMB, such as the CRISP3-bound form [39], are urgently needed.

## Supporting Information

S1 Fig Progression-free survival of prostatectomy (A) and ADT-treated (B) patients according to the MSMB immunostaining. Low MSMB expression: <20% of the cancer tissue was positively stained (with intensity 1–3). High MSMB expression: ≥20% of the cancer tissue was positively stained (with intensity 1–3). Mantel-Cox test was used to discover the *p*-values.
(TIF)

S2 Fig Progression-free survival of prostatectomy-treated patients according to (A) *MSMB*, (B) *NCOA4* and (C) *MSMB-NCOA4* expression. Expressions were measured with qRT-PCR. The median value was used as a cut-off point between high and low expression. The *p*-values were discovered by Mantel-Cox test.
(TIF)

S3 Fig RNA-sequencing data of expression of *MSMB*, *NCOA4* and *MSMB-NCOA4* in TCGA-cohort. TCGA-cohort included 301 prostate adenocarcinoma tumors. *NCOA4* was expressed at lower level than *MSMB*. *MSMB-NCOA4* expression was low compared to expression of *MSMB*. Y-axis represents normalized read count.
(TIFF)

S4 Fig Correlation of serum MSMB level with age of (A) PC cases and (B) controls. In figure A, age indicates age at diagnosis. In figure B, age indicates the time when blood sample was taken.
(TIF)

S5 Fig Correlation of serum MSMB level with PSA-levels. Correlation of serum MSMB level with free and total PSA levels of (A and B) cancer cases and (C and D) controls. (E) and (F) illustrates correlation between free to total PSA level and MSMB level in blood of (E) patients and (F) controls.
(TIF)

S6 Fig Progression-free survival of (A) prostatectomy-, (B) radiation- and (C) ADT-treated patients according to serum MSMB. The median level was used as a cut-off value for low and high level. Mantel-Cox test was used to discover the *p*-values.
(TIF)

S1 Table Characteristics of prostatectomy specimens used in IHC (n = 261) and qRT-PCR (n = 76). (DOCX)

S2 Table Characteristics of 99 needle biopsy specimens and 105 locally recurrent CRPCs used in IHC. (DOCX)

S3 Table Characteristics of the 369 PC cases of the cohort of serum and germ-line DNA. (DOCX)

S4 Table Association of serum MSMB levels and PC risk by the Gleason score and disease stage. (DOCX)
