# Correction: Microseminoprotein-Beta Expression in Different Stages of Prostate Cancer

**DOI:** 10.1371/journal.pone.0153732

**Published:** 2016-04-11

**Authors:** The PLOS ONE Staff

The twelfth author, Tapio Visakorpi, should also be listed as a corresponding author with the following email address: tapio.visakorpi@uta.fi.

The affiliation for the ninth author is incomplete. Johanna Schleutker is currently affiliated with Medical Biochemistry and Genetics, Institute of Biomedicine, University of Turku, Turku, Finland.

The publisher apologizes for the error.
