# Supplementary figures and images for: Microseminoprotein-Beta Expression in Different Stages of Prostate Cancer
Source: PLoS One. 2016 Mar 3;11(3):e0150241. doi: 10.1371/journal.pone.0150241 (PMC4777373; doi:10.1371/journal.pone.0150241)

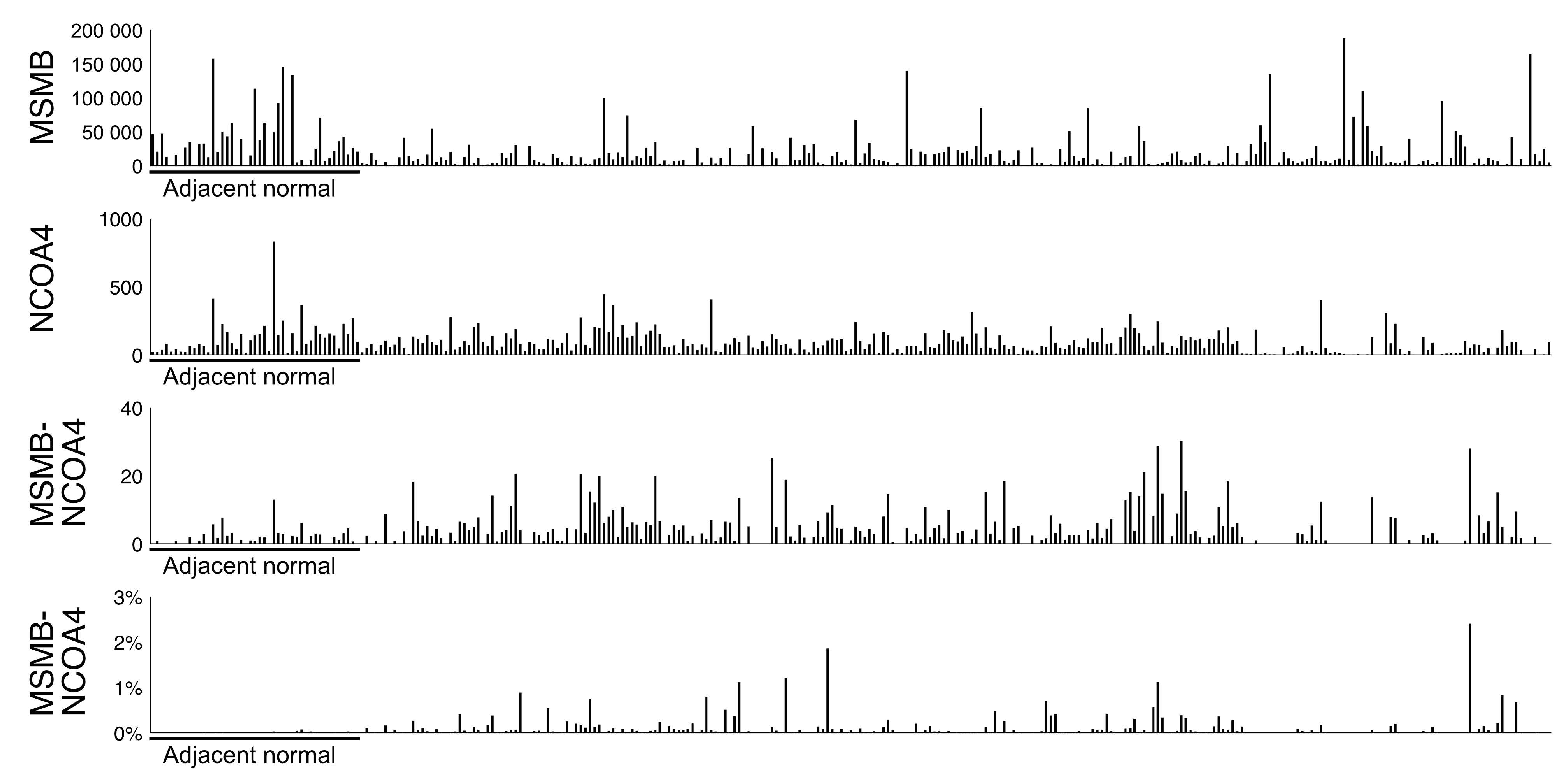

Supplement: S3 Fig — TCGA-cohort included 301 prostate adenocarcinoma tumors. NCOA4 was expressed at lower level than MSMB. MSMB-NCOA4 expression was low compared to expression of MSMB. Y-axis represents normalized read count. (TIFF) [file pone.0150241.s003.tiff]
