# Supplementary material for: Microseminoprotein-Beta Expression in Different Stages of Prostate Cancer
Source: PLoS One. 2016 Mar 3;11(3):e0150241. doi: 10.1371/journal.pone.0150241 (PMC4777373; doi:10.1371/journal.pone.0150241)
Supplement: S1 Table — (DOCX) [file pone.0150241.s007.docx]

S1 Table. Characteristics of prostatectomy specimens used in

IHC (n=261) and qRT-PCR (n=76).

| Characteristics |  |
| --- | --- |
| Prostatectomy specimens (IHC), n: | 261 |
| Gleason score, n (%) |  |
| ≤6 | 96 (36.8) |
| 7 | 128 (49) |
| ≥8 | 34 (13) |
| Unavailable data, n (%) | 3 (1.2) |
|  |  |
| pT-stage, n (%) |  |
| pT2 | 178 (68.2) |
| pT3 | 82 (31.4) |
| Unavailable data, n (%) | 1 (0.4) |
|  |  |
| Age, mean (range) | 62.9 (44-74) |
| Unavailable data, n (%) | 24 (9.2) |
|  |  |
| PSA ng/ml, median (IQR) | 10.3 (6.7-17.3) |
| Unavailable data, n (%) | 14 (5.4) |
|  |  |
| Prostatectomy specimens (qRT-PCR), n: | 76 |
| Gleason score, n (%) |  |
| ≤6 | 28 (37) |
| 7 | 30 (39) |
| ≥8 | 12 (16) |
| Unavailable data, n (%) | 6 (8) |
|  |  |
| pT-stage, n (%) |  |
| pT2 | 44 (57.9) |
| pT3 | 30 (39.5) |
| Unavailable data | 2 (2.6) |
|  |  |
| Age, mean (range) | 61.9 (47.4-71.8) |
| PSA ng/ml, median (IQR) | 8.4 (5.8-13.1) |
