# Supplementary material for: Microseminoprotein-Beta Expression in Different Stages of Prostate Cancer
Source: PLoS One. 2016 Mar 3;11(3):e0150241. doi: 10.1371/journal.pone.0150241 (PMC4777373; doi:10.1371/journal.pone.0150241)
Supplement: S2 Table — (DOCX) [file pone.0150241.s008.docx]

S2 Table. Characteristics of 99 needle biopsy

specimens and 105 locally recurrent CRPCs used

in IHC.

| Characteristics |  |
| --- | --- |
| Needle biopsy specimens, n: | 99 |
| Gleason score, n (%) |  |
| ≤6 | 24 (24) |
| 7 | 42 (42) |
| ≥8 | 33 (33) |
|  |  |
| Treatment, n (%) |  |
| Surgical castration | 22 (22) |
| Chemical castration | 65 (66) |
| Anti-androgen | 11 (11) |
| Combined androgen blockade | 1 (1) |
|  |  |
| Locally recurrent CRPC, n: | 105 |
| Treatment, n (%) |  |
| Orchiectomy | 46/105 (43.8) |
| LHRH analog | 29/105 (27.6) |
| Bicalutamide | 1/105 (1) |
| Orchiectomy and estrogen | 3/105 (2.9) |
| Combined androgen blockade | 22/105 (20.9) |
| Unavailable data | 4 (3.8) |
