# Supplementary material for: Microseminoprotein-Beta Expression in Different Stages of Prostate Cancer
Source: PLoS One. 2016 Mar 3;11(3):e0150241. doi: 10.1371/journal.pone.0150241 (PMC4777373; doi:10.1371/journal.pone.0150241)
Supplement: S3 Table — (DOCX) [file pone.0150241.s009.docx]

| Characteristics |  |
| --- | --- |
| Gleason score (biopsy), n (%) |  |
| ≤6 | 164 (44.4) |
| 7 | 114 (30.9) |
| ≥8 | 62 (16.8) |
| Unavailable data, n (%) | 29 (7.9) |
|  |  |
| Gleason score (prostatectomy), n (%) |  |
| ≤6 | 35 (30.2) |
| 7 | 66 (56.9) |
| ≥8 | 13 (11.2) |
| Unavailable data | 2 (1.7) |
|  |  |
| pT-stage, n (%) |  |
| pT2 | 80 (69) |
| pT3 | 33 (28.4) |
| Unavailable data, n (%) | 3 (2.6) |
|  |  |
| Treatment |  |
| Prostatectomy | 116 (31.4) |
| ADT | 102 (27.6) |
| Radiotherapy | 81 (22) |
| Brachytherapy | 20 (5.4) |
| Watchful-waiting | 33 (9) |
| Unavailable data | 17 (4.6) |
|  |  |
| cTNM, n (%) |  |
| T1NXMX | 126 (34.1) |
| T1NXM0 | 42 (11.4) |
| T1NXM1 | 1 (0.3) |
| T2NXMX | 63 (17.1) |
| T2NXM0 | 28 (7.6) |
| T2NXM1 | 4 (1.1) |
| T2N0M0 | 1 (0.3) |
| T3NXMX | 20 (5.4) |
| T3NXM0 | 41 (11.1) |
| T3NXM1 | 18 (4.9) |
| T3NXM1b | 1 (0.3) |
| T3N0M0 | 1 (0.3) |
| T3N1M1 | 2 (0.5) |
| Unavailable data | 21 (5.7) |

**S3 Table.** **Characteristics of the 369 PC cases of the cohort of serum and germ-line DNA.**
