# Supplementary material for: Microseminoprotein-Beta Expression in Different Stages of Prostate Cancer
Source: PLoS One. 2016 Mar 3;11(3):e0150241. doi: 10.1371/journal.pone.0150241 (PMC4777373; doi:10.1371/journal.pone.0150241)
Supplement: S4 Table — (DOCX) [file pone.0150241.s010.docx]

**S4 Table. The association of serum MSMB levels (quartiles) and PC risk by the Gleason**

**score and disease stage.**

|  | Q4 | Q3 | Q2 | Q1 | *p** | *p*** |
| --- | --- | --- | --- | --- | --- | --- |
|  | ≥31.41 ng/ml | 22.31-31.40 ng/ml | 17.71-22.30 ng/ml | ≤15.7 ng/ml |  |  |
| Gleason score |  | | | |  |  |
| <7 |  |  |  | |  |  |
| n (cases/ controls) | 36/220 | 54/214 | 33/239 | 41/228 | <0.001 | <0.001 |
| Odds ratio^†^ | 1.0 | 3.29 | 2.05 | 2.96 |  |  |
| 95% CI - lower | (ref.) | 1.86 | 1.12 | 1.63 |  |  |
| 95% CI - upper | (ref.) | 5.82 | 3.78 | 5.38 |  |  |
|  |  | |  | |  |  |
| ≥7 |  | |  | |  |  |
| n (cases/controls) | 52/220 | 42/214 | 42/239 | 40/228 | 0.003 | <0.001 |
| Odds ratio | 1.0 | 2.56 | 2.38 | 2.98 |  |  |
| 95% CI - lower | (ref.) | 1.41 | 1.31 | 1.6 |  |  |
| 95% CI - upper | (ref.) | 4.65 | 4.34 | 5.55 |  |  |
|  |  | |  | |  |  |
| Stage |  |  |  |  |  |  |
| M- |  |  |  |  |  |  |
| n (cases/ controls) | 30/220 | 24/214 | 36/239 | 25/228 | 0.004 | <0.001 |
| Odds ratio^†^ | 1.0 | 2.09 | 3.49 | 3.12 |  |  |
| 95% CI - lower | (ref.) | 0.994 | 1.729 | 1.469 |  |  |
| 95% CI - upper | (ref.) | 4.404 | 7.027 | 6.619 |  |  |
|  |  | |  | |  |  |
| M+ |  |  |  |  |  |  |
| n (cases/controls) | 14/220 | 4/214 | 3/239 | 5/228 | 0.65 | 0.014 |
| Odds ratio^†^ | 1.0 | 1.05 | 1.39 | 2.75 |  |  |
| 95% CI - lower | (ref.) | 0.13 | 0.23 | 0.52 |  |  |
| 95% CI - upper | (ref.) | 8.36 | 8.36 | 14.48 |  |  |

^†^Odds ratios are adjusted for age and total PSA (both linear and squared terms). * The *p*-value: whether

the dummie variables that represent the MSMB quartiles, taken together, are statistically significant.

** *p*-value for the association of MSMB modeled as a continuous variable. M-: local cancer;

M+: spread cancer.
